# Suitability of Mg-Nd and Mg-Zn Alloys to Obtain Biodegradable Structures for Bone Defects

**DOI:** 10.3390/jfb16110423

**Published:** 2025-11-12

**Authors:** Veronica Manescu (Paltanea), Aurora Antoniac, Maria Cristina Moraru, Iulian Antoniac, Cosmin Mihai Cotrut, Sebastian Gradinaru, Alexandra Iulia Dreanca, Bogdan Sevastre, Romelia Pop, Flaviu Alexandru Tabaran, George Mihail Vlasceanu, Mariana Ionita, Marius Manole

**Affiliations:** 1 Faculty of Material Science and Engineering, National University of Science and Technology Politehnica Bucharest, 313 Splaiul Independentei, District 6, 060042 Bucharest, Romaniaaurora.antoniac@upb.ro (A.A.); cosmin.cotrut@upb.ro (C.M.C.); 2 Faculty of Electrical Engineering, National University of Science and Technology Politehnica Bucharest, 313 Splaiul Independentei, District 6, 060042 Bucharest, Romania; 3 Faculty of Veterinary Medicine, University of Agricultural Sciences and Veterinary Medicine of Cluj-Napoca, 3-5 Calea Manastur, 400372 Cluj-Napoca, Romania; maria.moraru@usamvcluj.ro (M.C.M.); alexandra.dreanca@usamvcluj.ro (A.I.D.); bogdan.sevastre@usamvcluj.ro (B.S.); romelia.pop@usamvcluj.ro (R.P.); alexandru.tabaran@usamvcluj.ro (F.A.T.); 4 Academy of Romanian Scientists, 54 Splaiul Independentei, 050094 Bucharest, Romania; 5 Department of Medical-Clinical Discipline, Faculty of Medicine, Titu Maiorescu University, 67A Gheorghe Petrascu, 031593 Bucharest, Romania; sebastian.gradinaru@prof.utm.ro; 6 Department of General Surgery, County Hospital Ilfov, 050474 Bucharest, Romania; 7 Faculty of Medical Engineering, National University of Science and Technology Politehnica Bucharest, 1-7 Gheorghe Polizu, 011061 Bucharest, Romania; george.vlasceanu@upb.ro (G.M.V.); mariana.ionita@polimi.it (M.I.); 8 Advanced Polymer Materials Group, National University of Science and Technology Politehnica Bucharest, 1-7 Gheorghe Polizu, 011061 Bucharest, Romania; 9 Department of Prosthetics and Dental Materials, Faculty of Dentistry, Iuliu Hatieganu University of Medicine and Pharmacy, 8 Victor Babes, 400012 Cluj-Napoca, Romania; marius.manole@umfcluj.ro

**Keywords:** magnesium alloys, microstructure, corrosion, biocompatibility

## Abstract

Mg-based alloys are one of the most promising materials used in regenerative medicine for bone tissue engineering. Considering the increasing prevalence of a continuously aging population, as well as the high incidence of accidents and bone cancers, it is crucial to explore biomaterials that can serve as bone substitutes. After carefully analyzing the literature in the introduction section, we proposed two Mg-based alloys as suitable for obtaining biodegradable structures for bone defect treatment. To achieve trustworthy results, the alloys’ microstructure was investigated using microscopic techniques coupled with energy-dispersive spectroscopy and X-ray diffraction. The obtained results were comparable with those described in references on similar Mg alloys. Then, the mechanical compression properties were highlighted, and the *in vitro* corrosion behavior proved that Mg-Zn exhibited a reduced corrosion rate compared to the Mg-Nd alloy, as tested using electrochemical methods. However, the *in vivo* tests showed good biocompatibility for both magnesium alloys. In conclusion, both alloys are suitable for use as potential bone substitute applications, but it must be taken into consideration that Mg-Zn alloys present lower biodegradation and mechanical properties. For future investigations, we aim to develop bone substitutes made from these materials, specifically designed for small bone defect treatment and with patient-adapted geometry. Due to the differences mentioned above, various designs will be tested.

## 1. Introduction

Nowadays, modern lifestyle changes correlated with an increased life duration have made orthopedics one of the most challenging medical domains. Due to car or motorcycle accidents or even trauma caused by sports practice, there is an important need to address the bone defects that usually occur [1,2]. However, in medical science, it is stated that bone injuries are generated also caused by tumors, infections, congenital issues, and even surgeries [3,4]. Small bone defects exhibit the ability to spontaneously heal, but large bone defects usually require supplementary care, and various solutions are applied to alleviate the patient’s suffering [5,6,7]. A very realistic and generally accepted classification of bone defects was made by Schemitsch [8], who defined small bone defects as those with a size below 2 cm and a maximum of 50% cortical loss circumference. In his opinion, intermediate defects have a size between 2 and 6 cm correlated with a circumference loss higher than 50%. Last but not least, the largest defects with a size greater than 6 cm are considered a major health concern, being very serious and leading in some cases to limb amputation and to an important decrease in the patient’s quality of life with a high social burden [9].

To fix these bone defects, one can identify the continuous need to develop new and innovative solutions for bone substitutes, which is a major concern for scholars. The use of the autograft was long considered the gold standard, but problems such as limited patient bone quantity and donor site morbidity prevented it from being applied on a large scale [10,11]. Another solution met in practice consists of using allografts or xenografts, which are harvested from human or animal donors, respectively. Unfortunately, these interventions are also associated with a high risk of graft rejection or disease transmission, with unanticipated evolution due to bacteria or pathogen adaptation from passing through the organisms of different individuals or species [12,13,14,15]. Another major drawback of the grafting techniques is their high cost, which is associated with a continuous increase in graft demand that has led to the necessity of developing synthetic bone substitutes.

Georgeanu et al. [1] underlined the most important attributes of the ideal biomaterial used as a bone substitute. The authors stated that it must exhibit osteoinductive, osteogenic, and osteoconductive properties. An adequate bone substitute should stimulate both angiogenesis and neurogenesis, and it must not induce any teratogenic, allergic, antigenic, carcinogenic, or other adverse reactions. In addition, its surface must be rough and exhibit good wettability to facilitate cell adhesion and proliferation. Also, the mechanical properties of an ideal biomaterial should have values close to those of the human bone, and last but not least, it must not be degraded by clinical handling and must offer the possibility of being easily sterilized. Other characteristics that have lately been underlined are bone substitute bioactivity and biodegradability, which concomitantly help the bone regeneration process with the bone substitute’s gradual dissolution [16,17]. One of the most modern solutions should incorporate all these requirements.

The synthetic materials that are today used as bone substitutes are classified as ceramics, polymers, or composites. Bongio et al. [16] identified the first material generation as inert and tolerant, providing material fibrous encapsulation as a host tissue response. The second generation constitutes responsive biomaterials such as bioglasses, being characterized by degradation and osteoconductivity, while the third generation comprises inductive materials that are able to develop a strong link between natural bone and substitute, as well as to enhance osteoblast production simultaneously with blood vessel formation and, where necessary, nerve regeneration.

From the first generation of biomaterials, one can name thermoplastic polymers and ceramics including zirconia or alumina with important drawbacks such as inadequate mechanical properties and the lack of ability to connect with the hard tissue of the host [6,16,17,18,19]. From the second generation, bioglasses are considered osseous replacements with high biocompatibility and increased osteoconductivity. When adequate processing conditions are chosen, a porous structure can be achieved. This is, in particular, beneficial for bone development. Usually, bioglasses are not associated with inflammatory reactions or systemic toxicity, being resorbable in a certain amount of time. The degradation rate could be controlled through adjustments in chemical composition. Some of the most common glasses in practice are those based on silica, borate, or phosphate [20,21,22]. The last ones are especially characterized by a bioactive character, and many studies [23,24,25] proved that they could form a strong connection with natural bone. The main disadvantage of glasses is that they are brittle, have low mechanical strength, and decreased fracture resistance. Regarding the third biomaterial group, one can mention different types of calcium phosphate such as β-tricalcium phosphate (β-TCP), hydroxyapatite (HA), or a combination between them in the shape of biphasic calcium phosphate (BCP) ceramic [26,27]. These types of materials are characterized by increased osteoinduction that can be enhanced through different methods such as bone-forming cells, growth factors, and bioactive protein addition [28,29,30]. Some studies [31,32] showed that the slow release of vascular endothelial growth factor (VEGF) from BCP determined an increase in blood vessel formation. Polymers represent another solution adopted by practitioners because they are very versatile regarding their physical or chemical properties [33,34,35]. In the case of these biomaterials, an important drawback, which can be immediately foreseen, consists of the fact that they are inert, and osteoinduction must be controlled through different approaches because hydrogels used as simple templates do not sustain cell adhesion and viability in a high percentage of cases [36,37]. If polymers are associated with bioactive factors, highly bioactive bone substitutes can be obtained with adequate degradation kinetic process but with poor mechanical properties such as reduced stiffness and strength.

**Figure 1:**
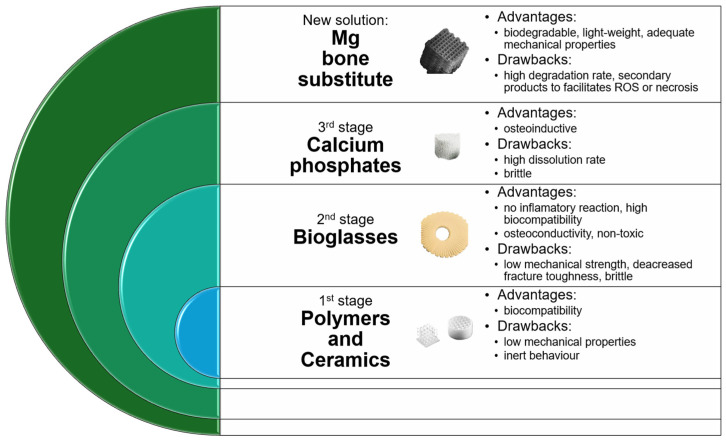
Main bone substitutes used in clinical practice and new solutions with advantages and drawbacks.

A response to the current limitations, which was adopted in many studies, consists of using biodegradable metal in bone substitute manufacture (Figure 1). From this class of biomaterials, magnesium (Mg) and its alloys stand out as the most promising candidates due to their excellent physical, chemical, and mechanical properties. This biodegradable and lightweight metal can be used as a bone substitute in two forms: as a powder to fill the bone gap directly and as a block inserted into the defect. One of the most vital mechanical properties of Mg is its Young’s modulus, which is between 41 and 45 GPa, a value considered very close to that of human bone (3 ÷ 20 GPa) [38,39,40]. Regarding the yield strength of Mg (65 ÷ 100 MPa), this mechanical quantity is slightly lower than that of natural bone (104 ÷ 121 MPa). However, one can immediately notice that the stress shielding effect is estimated to occur very rarely due to similar values between Mg and human bone in terms of their mechanical properties. Another key feature in bone substitute design is the degradation process of the alloys. In the case of Mg, the main secondary products are magnesium hydroxide (Mg(OH)_2_) and hydrogen (H_2_), which should be carefully controlled to avoid generating harmful effects such as reactive oxidative species formation and necrosis due to hydrogen pockets that usually occur in the vicinity of the Mg bone substitute [41]. In addition, the concentration of Mg^2+^ ions must be kept in the following interval: 5 ÷ 10 mmol/L to avoid adding supplementary toxicity [42,43,44]. Some studies [45,46,47] showed that Mg^2+^ ions could exhibit a beneficial effect on osteoblast differentiation due to the medium alkalinization that reduces osteoclast activity and accelerates mineralization, concomitantly with a stimulation of the calcitonin gene-related peptide (CGRP) that is well-known to sustain the differentiation of stem cells into osteoblasts [48,49,50]. Usually, the most common solutions to control the biodegradation rate of Mg-based alloys consist of Mg alloying with different chemical elements to obtain new Mg-based alloys or developing biocompatible coatings based on ceramics, polymers, or composites for existing Mg-based alloys [6,7,51,52].

The main binary Mg-based systems used in developing medical applications are Mg-Ca, Mg-Zr, Mg-Zn, Mg-Ag, and Mg- rare earths (REs) such as Mg-Nd and Mg-Y [6,39,53,54,55,56]. We will discuss only the importance of alloying Mg with neodymium (Nd) and zinc (Zn) from all of these systems because they are directly related to our experimental investigation. Neodymium is a rare earth and is usually added to improve the alloy’s mechanical properties and increase the corrosion resistance due to the modifications induced in the material microstructure [57]. Different studies demonstrated that the Nd percent should be carefully selected because it forms a phase (Mg_3_Nd) that can lead to an increase in the corrosion process [58,59,60]. In addition, a shielded oxide layer was noticed at the surface of the Mg-Nd binary alloy. Regarding the alloying of Mg with Zn, the tolerance limit of Zn in Mg-Zn alloys was established to be around 3 wt.% [61,62]. In the Mg-Zn alloys, the Zn percent should be controlled because it plays an important role in biocorrosion by reducing the emission of hydrogen, which typically occurs during the Mg degradation process. Otherwise, Zn, being a trace element in the human body, is highly biocompatible and is present in muscles and bones. Kirkland mentioned that the corrosion rate increases when the Zn content varies from 1 to 3 wt.% due to crack formation [62].

Mg-based alloys have addressed almost all the limitations of traditional bone substitutes (e.g., second surgery, stress shielding, inert character) because they degrade and are replaced by new bone, have a Young’s modulus with values very close to those of human bone, prevent the stress shielding effect, and last but not least, promote bone regeneration.

The current study aims to evaluate two binary Mg-based alloys from the systems Mg-Nd and Mg-Zn to be applied as bone substitutes in different shapes. We will investigate their microstructural characteristics based on optical microscopy, scanning electron microscopy (SEM) coupled with energy dispersive spectroscopy (EDS), and X-ray diffraction (XRD). The mechanical properties of the two binary alloys will be determined based on the compression test. Also, the corrosion of the experimental magnesium alloys will be assessed with electrochemical analysis in simulated body fluid (SBF). The biocompatibility will be evaluated by *in vivo* testing on rats, followed by histological analyses and microcomputed tomography (μCT).

## 2. Materials and Methods

Two magnesium-based alloys, from the binary systems Mg-Nd and Mg-Zn, were chosen to be investigated and tested to establish if these are suitable for bone substitutes in orthopedics. The Mg-Nd alloy was produced using sand mold and chill casting, while the Mg-Zn alloy was fabricated via the stir casting method. Both alloys were synthesized from high-purity raw materials (Mg, Nd, Y, Zn, Ca, and Mn—99.99% purity). Their chemical composition is provided in Table 1. Experimental samples for material characterizations were prepared as parallelepipedal blocks with edge lengths of 10 mm, obtained through mechanical cutting.

### 2.1. Microstructural Characterization of Experimental Samples

The microstructural characterization of the samples was conducted using a Nikon Eclipse MA100N inverted microscope (Nikon Metrology Europe NV, Leuven, Belgium) and an ESEM Quattro S microscope, which has an EDS UltraDry 60M module (Thermo Fisher Scientific Inc., Waltham, MA, USA). For the optical observations, the Mg-based alloy sample surface was metallographically prepared by chemical etching using a Nital reagent. The phase identification was performed using a PANALYTICAL x-Pert PRO diffractometer (Malvern Panalytical, UK) equipped with CuKα radiation. The acquired information was processed in PDXL Software 1.2.0.1 (ICDD 1999).

### 2.2. Mechanical Properties

The compression test was conducted based on a Walter + Bai LFV 300 (Walter + Bai AG, Löhningen, Switzerland) device in accordance with the ASTM E9-09 standard [63]. The measurements were performed as a function of displacement control with a speed of 5 mm/min. The ending criterion was set when an applied force decrease of about 75% from the maximum value was reached. After the compression tests were finished, the stress–strain diagram was obtained as the main result.

### 2.3. In Vitro Corrosion Behavior of the Experimental Samples

The Mg-Nd and Mg-Zn corrosion process was investigated based on electrochemical analysis. In the case of the above-mentioned method, we chose simulated body fluid (SBF) because this electrolyte has a chemical and ionic composition similar to that of human blood plasma [64]. The SBF was prepared in our laboratory using the Kokubo protocol [65] and characterized by a physiological pH of 7.4.

The electrochemical analysis was performed based on PARSTAT 4000 Potentiostat/Galvanostat equipment (Princeton Applied Research, Oak Ridge, TN, USA) with a three-electrode cell according to ASTM G5-94 (2011) standard [66]. The testing protocol used to determine the corrosion parameters was similar to that described in [57,67,68]. Briefly, based on a CW-05G (Jeio Tech, Seoul, Republic of Korea) heating and recirculation bath, the electrochemical measurements were performed at 37 °C. Three samples prepared from the two Mg alloys, respectively, were inserted into a dedicated Teflon support to expose only a surface area of 1 mm^2^ to the SBF action. The Tafel dependencies were determined in a potential range of ± 250 mV vs. EOC (potential rich at quasi-equilibrium) at a speed of 1 mV/s. The EOC was measured for 1 h. The main corrosion parameters were computed based on potentiodynamic variations plotted in Versa Studio 2.63.3 Software.

### 2.4. In Vivo Animal Study

#### 2.4.1. Surgical Procedure and Histopathological Analysis Route

The amount of material for *in vivo* tests was measured precisely employing a calibrated analytical balance (Kern ABJ 220-4NM, Merk, Germany). Specifically, 140 mg of material was applied subcutaneously, and 40 mg was used to fill the bone defects. Before implantation, both Mg-Nd and Mg-Zn underwent a wet sterilization procedure by autoclaving with a Trade Raypa Steam Sterilizer (R. Espinar S.I., Spain, A.E-75 Dry) at 105 °C and 1 Bar for 5 min.

Seventy-two specific pathogen-free (*SPF*), 40-week-old female Sprague Dawley rats originated from the “Cantacuzino” National Institute for Medical-Military Research and Development (Bucharest, Romania) were involved in the study. Animals were housed under SPF laboratory conditions with free access to food and water.

For the subcutaneous compatibility investigation, 32 animals were used, 16 for each material. Four animals from the two alloy-based groups were euthanized at 1, 2, 4, and 8 weeks.

The bone implantation was conducted on 40 animals, randomly allocated into two experimental groups (*n* = 20), of which samples of Mg-Nd and Mg-Zn alloys were used. Five animals from each group were euthanized at the same intervals of 1, 2, 4, and 8 weeks for the collection of the bone tissue. The rationale for choosing an investigation time of a maximum of 8 weeks to collect bone tissue was in accordance with many literature studies that involved bone defects in Sprague Dawley rats and evaluated local and systemic biocompatibility in both soft and hard tissues, rather than long-term bone regeneration and complete material degradation. Therefore, the selected time point corresponds to an acute phase assessment, in accordance with ISO 10993-6 [69] recommendations for biological evaluation of medical devices [70,71]. This time was considered sufficient for the natural bone healing process with substantial but not complete remodeling or bone formation [72]. The study of Nowak et al. [73] recommended 12 weeks for complete healing. However, an 8-week approach is a more adequate choice when biodegradable metals such as Mg alloys are used, as it allows for the study of early bone mineralization and new bone formation in conjunction with alloy degradation [74]. This process is synchronically connected with its replacement by animals’ natural hard tissue, and by choosing 8 weeks as a correct time, an *in vivo* evaluation before complete bone maturation could be conducted [74,75,76]. Most of the research studies opted for a maximum of 8 weeks for the investigation time based on practical considerations because at this point, important effects of the bone substitute are usually still present [77,78]. In addition, at the 1-week time point, early molecular and cellular mechanisms such as the onset of osteogenesis and inflammation could be studied [79]. At 2 weeks, osteogenesis and angiogenesis are enhanced due to the action of Mg^2+^ ions [6,80,81], while at 4 weeks, early bone formation and osteoblast activity can be monitored [79].

All procedures were approved by the Bioethics Committee of the University of Agricultural Sciences and Veterinary Medicine Cluj-Napoca (Ethical approval 386/15 June 2023), and the project was approved by the regional state veterinary authority DSVSA-CN (Project authorization no. 382/25 August 2023).

General anesthesia was induced by intraperitoneal injection of ketamine (80 mg/kg) and dexmedetomidine (0.5 mg/kg). Analgesia was provided using intramuscular tramadol (15 mg/kg). Corneal lubrication was applied to prevent desiccation. Anesthesia depth was monitored continuously, and body temperature was maintained with a heating pad. Atipamezole was administered at the end of the procedure to reverse dexmedetomidine.

**Figure 2:**
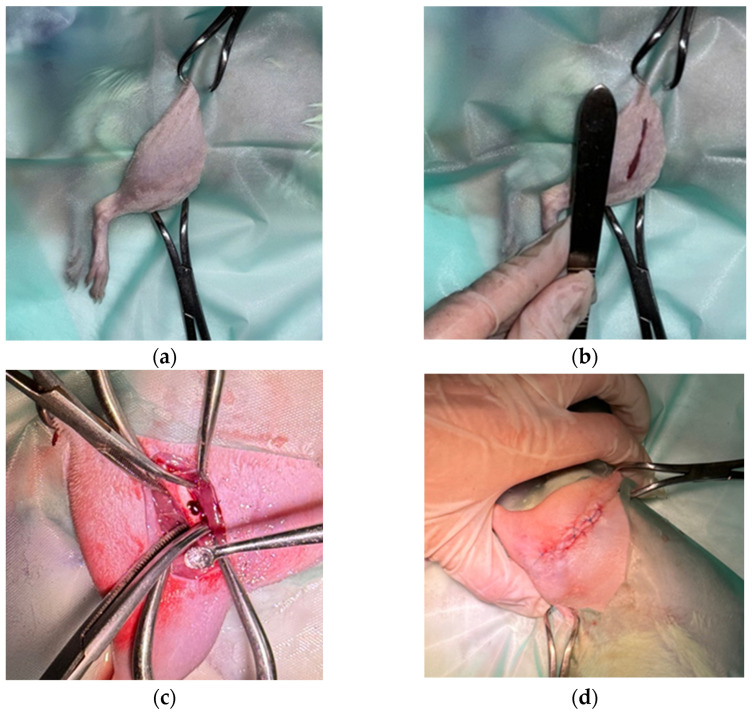
Surgical images during the medical procedure performed on rat bones: (**a**) Placement for surgery; (**b**) Incision site; (**c**) Bone defect and Mg alloy samples; (**d**) Sutured surgical wound.

Under aseptic conditions, a cranio-lateral skin incision was made over the right femur. Blunt dissection was carried out between the *biceps femoris* and *fascia lata* to expose the femoral diaphysis without damaging the periosteum. A standardized cortical bone defect, 3 mm in length, was created using a 1.2 mm diamond burr, with continuous saline irrigation to prevent thermal injury. Then, the defect was filled with either Mg-Nd or Mg-Zn powder. The wound was closed in three layers using 4-0 absorbable monofilament sutures for fascia and subcutaneous tissue, and 4-0 non-absorbable nylon in an interrupted pattern for the skin (Figure 2).

**Figure 3:**
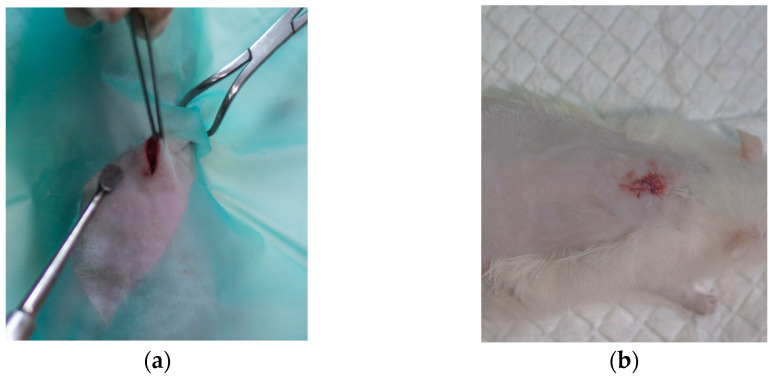
Surgical images during the medical procedure performed on the rats’ skin: (**a**) Subcutaneous placement of Mg alloy samples; (**b**) Surgical site.

For subcutaneous implantation, the rats were positioned in sternal recumbency. One small dorsal incision was made under aseptic conditions. In each group, 140 mg of powdered magnesium alloy (Mg-Nd or Mg-Zn) was inserted into the subcutaneous space using a Volkmann spoon. The skin was closed with 4-0 nylon sutures in a simple interrupted pattern (Figure 3).

Post-operative supportive therapy included subcutaneous 0.9% NaCl for 2–3 days. Analgesia was maintained with tramadol (15 mg/kg IM) and oral metamizole (150 mg/kg, dose-adjusted based on the rat grimace scale). Enrofloxacin (10 mg/kg SC) was administered once daily for 5 days as antibiotic prophylaxis. The rats were euthanized at 1, 2, 4, and 8 weeks post-implantation. Skin and femoral bone samples were collected for further evaluation.

After harvesting, skin and bone tissues were fixed in 10% neutral-buffered formalin for 48 h. For bone samples, fixation was followed by 24 h decalcification in a rapid solution and storage in 70% ethanol. All samples were dehydrated in graded ethanol, cleared in xylene, and embedded in paraffin at 58 °C. Sections of 2 µm were cut using a rotary microtome. Slides were deparaffinized in xylene, rehydrated through descending ethanol concentrations, and rinsed in tap water. Standard hematoxylin–eosin (HE) staining was applied: hematoxylin (5 min), rinsing (5 min), eosin (2 min), followed by dehydration in graded ethanol and clearing in xylene. Coverslips were mounted with Permount^TM^ toluene-based synthetic resin.

Histological evaluation was performed under an Olympus BX51 microscope (Olympus Corporation, Tokyo, Japan). Images were acquired using an Olympus UC30 (Olympus Corporation, Tokyo, Japan) digital camera, processed with Olympus cellSens imaging software version 3.1.

Subcutaneous implant-associated lesions were scored according to ISO 10993-6:2021 [69] guidelines for the biological evaluation of medical devices. The average score per parameter and the overall biological response were calculated based on three representative microscopic fields per sample, from three independent, blinded observations. The scoring criteria included the evaluation of parameters such as inflammatory cell infiltration, fibrosis, necrosis, neovascularization, and new bone formation for bone samples, and epithelial integrity, inflammatory response, and fibrosis. Each criterion was graded semi-quantitatively from 0 (no reaction) to 4 (severe reaction).

#### 2.4.2. Micro-Computed Tomography (µCT) Acquisition and Analysis

Femoral specimens were imaged on a Bruker SkyScan 1272 (Kontich, Belgium) at 70 kV and 140 µA, with a 0.5 mm aluminum filter. Projections (2452 × 1640 px) were collected every 0.2° through 360° using 1400 ms exposures and three-frame averaging. Data were reconstructed in NRecon 1.7.1.6 software with ring- and beam-hardening corrections to an isotropic voxel size of 3.5 µm. A volume of interest (VOI) was drawn slice by slice in CTAn to match the original oval cortical window in the mid-diaphysis. The outline followed the adjacent cortex, excluding mineral that migrated into the medullary canal or formed beyond the defect. Irregular margins due to remodeling and variable cortical thickness were accommodated.

**Figure 4:**
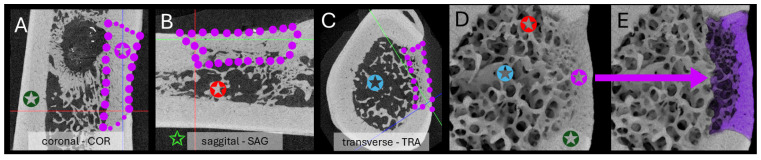
Illustration of manually defined limits of volume of interest (VOI) in orthogonal planes ((**A**) coronal COR, (**B**) sagittal SAG, (**C**)) transverse TRA extracted from DataViewer 1.5.4.6 software (magenta dots), as part of the segmentation process—exemplified for sample Mg-Nd_W8. The tomogram slice highlights key anatomical and pathological features, including intact femoral cortex (✪), medullary channel (✪), mineralized tissue within both the medullary cavity (✪) and the cortical defect (✪), and surrounding soft tissue (**✩**). The (**D**,**E**) subsets depict a TRA cross-section of the femur tomogram (CTVox 3.3.0.0 software) at the Mg-Nd_W8 (8 weeks) implantation site. The segmented bone volume (BV) selected for analysis is shown in magenta, representing the region of interest within that specific slice.

Within the VOI, thresholding rendered bone voxels white (bone volume, BV); all remaining voxels were treated as porosity and omitted from BV calculations (Figure 4).

Bone mineral density (BMD) was measured ex vivo from micro-CT scans of explanted samples collected at 1, 2, 4, and 8 weeks post-implantation. Values (g/cm^3^) were calculated in CTAn 1.17.7.2 software using calibrated attenuation coefficients, with hydroxyapatite (CaHA powder) embedded epoxy resin calibration rod phantoms (⌀ 4 mm) serving as reference standards.

## 3. Results and Discussion

### 3.1. Microstructural Characterization of the Mg-Based Alloys

#### 3.1.1. Optical Microscopy

**Figure 5:**
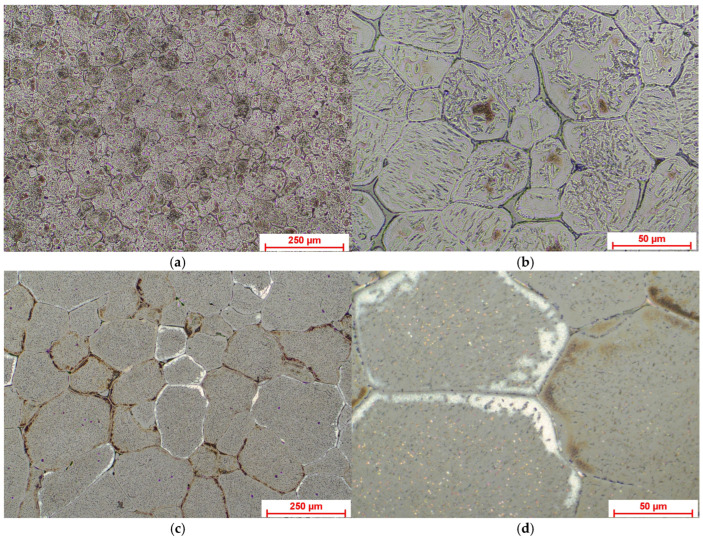
Optical microscopy images showing the microstructural aspects of Mg-Nd alloy: (**a**) magnification 10×; (**b**) magnification 50×; and Mg-Zn alloy: (**c**) magnification 10×; (**d**) magnification 50×.

The Mg-Nd and Mg-Zn sample surfaces were metallographically prepared for optical observations by chemical etching with Nital 2.5%. Figure 5 presents the optical images obtained for both Mg-based alloys.

In the case of the sample made of Mg-Nd, one can clearly observe polyhedral grains of α-Mg, in which lamellar and globular morphologies are found. The grains are big and exhibit non-uniform size, with a reduced degree of intergranular precipitation. The lamellar compounds tend to form groups into the grains, and this fact induces better mechanical properties. Our findings confirm the fact that Mg-based alloys containing rare earths, like Nd, exhibit good mechanical strength and corrosion behavior [82,83,84,85].

For the Mg-Zn experimental samples, the optical microscopy images show that the material microstructure presents uneven and larger α-Mg polyhedral grains compared to the Mg-Nd experimental samples. Inside the grains, spheroidal and irregularly contoured compounds are visible, which will be further investigated based on SEM-EDS analysis. Microstructure refinement and improvement of mechanical properties are usually performed by adding different alloying elements such as Mn and Zn. Mn is usually used to reduce the grain size concomitantly with an improvement in the tensile strength of the alloy [86], while Zn has beneficial effects on increasing the tensile strength and corrosion resistance [87,88,89].

#### 3.1.2. X-Ray Diffraction Analysis (XRD)

**Figure 6:**
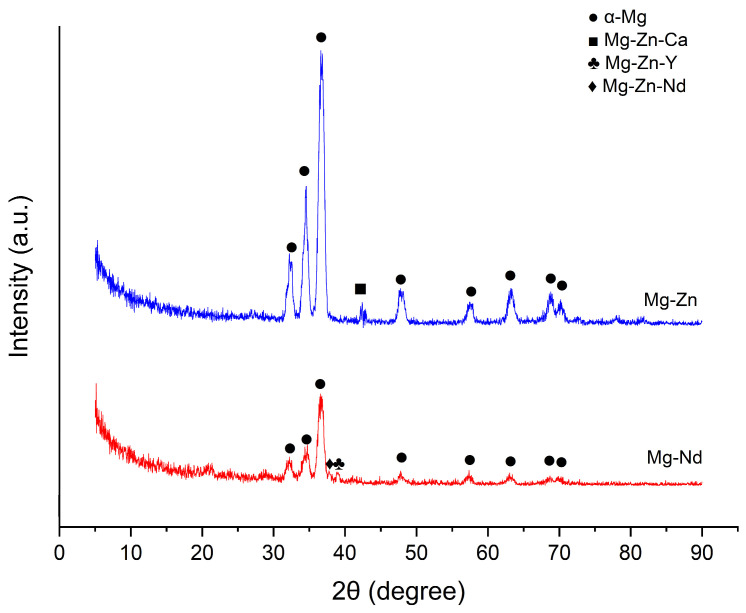
X-ray diffraction patterns of the Mg-Nd and Mg-Zn alloys.

As one can observe from Figure 6, in the case of Mg-Nd alloy, the identified peaks represent α-Mg and ternary phases (Mg-Zn-Nd and Mg-Zn-Y). Yang et al. [90] noticed in the case of Mg-4.5Zn-*x*Nd (*x* = 0, 1, and 2 wt%), which contains Zn and Nd as in our investigated magnesium alloys, the presence of the ternary Mg-Zn-Nd phase. They identified this phase as T-phase (Mg_4_Zn_2_Nd), which exhibits a *c*-centered orthorhombic crystal structure, as presented in [91,92]. In the case of our Mg-Nd alloy, we found that the ternary phase corresponds to the T-phase, as it was noticed in the literature [90,93].

Additionally, in accordance with the research performed by Zengin and Turen [94], considering the fact that the Y concentration is about 0.3 wt%, we can observe a supplementary ternary phase, Mg-Zn-Y, denoted in the literature as I-phase (Mg_3_Zn_6_Y). Other phases reported are W-phase (Mg_3_Zn_3_Y_2_) and X-phase (Mg_12_ZnY) [95,96,97], but these were not existent in our Mg-Nd alloy, being generally associated with an increased ratio of Y and Zn [98,99].

Regarding the other investigated alloy, Mg-Zn, with a Zn mass percent of 1.4 wt%, the diffraction peaks are associated with α-Mg and Mg-Zn-Ca ternary phase. The existence of the ternary Mg-Zn-Ca (Mg_6_Zn_3_Ca_2_) and a supplementary binary Mg-Ca (Mg_2_Ca) phases was evidenced by the studies of Farahany et al. [100], Jiang et al. [101], and Schäublin et al. [102] and was conditioned by a Ca content more elevated than 0.5 wt%. In addition, for a Zn/Ca ratio with values higher than 1.23, the α-Mg and Mg-Zn-Ca ternary phases are usually identified in the XRD spectra [103,104]. For our samples, we did not have evidence of the presence of the binary phase Mg-Ca because the value of Zn/Ca is about 4.66, and the Ca content is 0.3 wt%. Some studies [105,106,107] showed the existence of a supplementary α-Mn phase precipitate, but in our case, this phase was not identified. This observation is in agreement with the analyses performed by Cho et al. [108] and Bakhsheshi-Rad [88], who reported the absence of Mn intermetallic compounds and α-Mn phase in the case of Mg-4Zn-0.5Ca-(0, 0.4, 0.8 wt%)Mn and Mg-2Ca-0.5Mn-(2-7 wt%)Zn, respectively.

The SEM findings will fulfill the investigation presented above very well by clarifying the chemical and physical processes that occurred during the material manufacturing steps, while XRD clearly showed the phases present in the microstructure.

#### 3.1.3. Scanning Electron Microscopy

**Figure 7:**
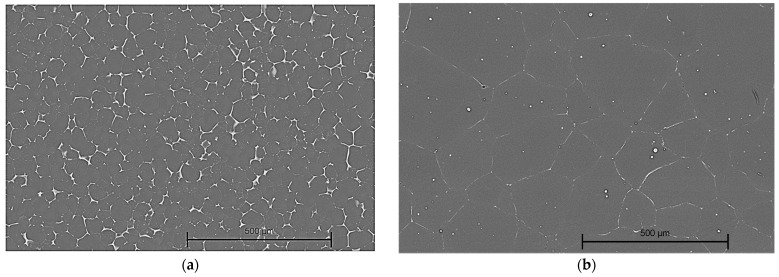
SEM images of samples made of: (**a**) Mg-Nd alloy, magnification 100×; and (**b**) Mg-Zn alloy, magnification 100×.

It is well-known that material microstructure, grain shape, and size significantly influence alloy properties such as corrosion resistance, plasticity, and strength. In the case of the Mg-Nd and Mg-Zn samples, we obtained microstructure images similar to those from optical microscopy (Figure 7).

Based on EDS analysis, in the case of both investigated magnesium alloys, the main chemical elements were identified (Table 2). This fact is directly correlated with the chemical composition of the material described in Table 1.

**Figure 8:**
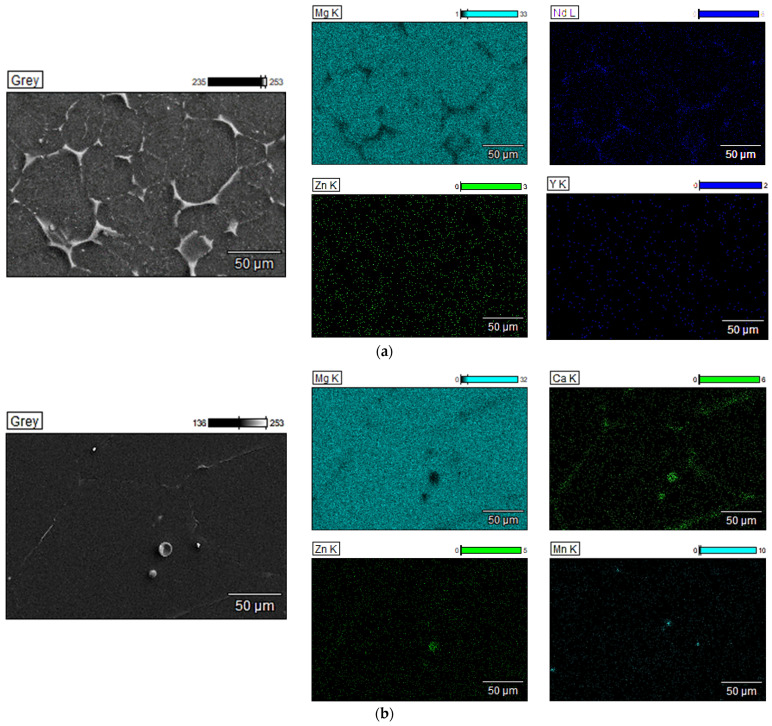
EDS elemental mapping results (magnification 500×) for (**a**) Mg-Nd alloy; (**b**) Mg-Zn alloy.

**Figure 9:**
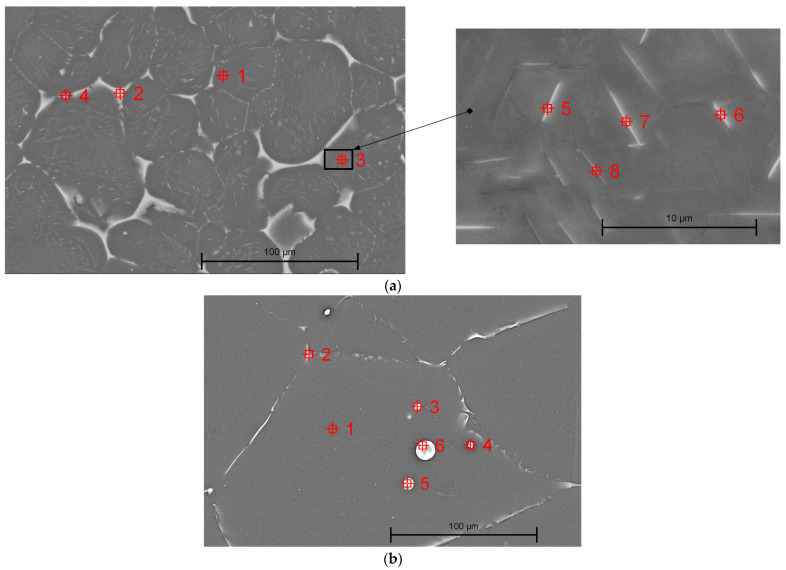
EDS analysis conducted for identification of phases’ chemical composition: (**a**) Mg-Nd alloy—magnification 500× (Point 3—Inset (**a**) magnification 6000×); (**b**) Mg-Zn alloy—magnification 500×.

In addition, in Figure 8a, the EDS mapping images of Mg-Nd sample with the chemical element spatial distribution are presented. It can be noticed that the alloy matrix consists mainly of Mg and Nd, while some traces of Zn and Y can be also observed (point 1, Table 3). In [109,110,111], it is stated that when the Nd quantity is higher than 2 wt.%, only 1.4 ± 0.1 wt.% Nd is stable, proving saturation of the solid solution of Nd in the Mg matrix. For the investigated Mg-Nd alloy, the supplementary Nd quantity can be localized at the grain boundary (Figure 8a and Figure 9a and Table 3). The EDS analysis confirms that a phase Mg-Zn-Nd is present at the grain boundary (point 2, Table 3), and in the lamellar compounds identified based on optical microscopy and SEM in α-Mg matrix (points 3, 5, 6, 7, and 8, Table 3). Regarding the white compound placed at the grain boundary, an intermetallic phase Mg-Zn-Y was detected (Table 3, point 4).

Elkaiam et al. [112] analyzed the microstructure of Mg-5%Zn-0.13%Y-0.35%Zr, adding up to 3% Nd. SEM images evidenced that α-Mg matrix was prevalent in the alloy, and the Nd additions were linked to precipitations around the magnesium grains. The authors noticed that modifying the Nd concentration led to different stoichiometry of Mg-Zn-Nd ternary phases that were localized based on EDS analysis at the grain boundaries. In addition, in our case, the Y concentration was higher, leading in the Y presence in the α-Mg matrix. Gong et al. [113] identified in Mg-2Zn-0.5Y-1.0Nd a quasi-crystalline phase rich in Zn with a chemical formula of Zn_60_Mg_30_RE_10_, in accordance with other studies [94,114].

In the case of the second investigated alloy, Mg-Zn, SEM images showed an α-Mg matrix with secondary phases localized mainly at the grain boundaries in a strip-like structure. EDS analysis revealed a chemical composition (Figure 8b) for these secondary phases with elements such as Mg, Ca, and Zn, and a localized distribution of Mn.

Figure 9b and Table 3 present some representative points set in different zones of the Mg-Zn microstructure. Point 1, Table 3 is localized inside the grain and exhibits mainly α-Mg as well as Zn, Ca, and Mn that are uniformly distributed into the whole matrix. Regarding the grain boundary investigation, we set point 2, Table 3, which was characterized by the presence of Mg, Zn, and Ca. The small round compounds (points 3 and 4, Table 3) are rich in Mn, as reported in the literature [106]. Regarding the large round compounds (points 5 and 6, Table 3), the EDS analysis revealed a similar chemical composition of Mg, Zn, and Ca as that found at the grain boundary. The XRD analysis supports this observation. In good agreement with our results extracted from EDS analysis and XRD investigation, some studies [100,102,106] proved the formation of Mg_6_Zn_3_Ca_2_ phase instead of MgZn because the diffusivity of Ca in Mg is superior to that of Zn in the same matrix.

SEM investigations are of utmost importance when the material microstructure is analyzed because they can clarify the observations obtained after optical microscopy.

### 3.2. Mechanical Characterization of the Mg-Based Alloys

The manufacturing technology of Mg-based alloys has a significant impact on the elastic modulus and compressive strength of the material. For instance, in the case of our cast alloys, when compared to the other technologies, less optimized mechanical properties could be achieved because the coarse grain structure leads to lower values of Young’s modulus and compressive strength [115,116]. The wrought processes typically result in a reduced grain size by promoting the precipitation process and enhancing the aforementioned mechanical properties [117,118]. Regarding the additive manufacturing technology, high-temperature working conditions are directly linked to differences in mechanical performance, while low-temperature methods lead to higher mechanical properties [119]. For Mg-based alloys manufactured through powder metallurgy, the compressive strength value is influenced by the powder content and production conditions [120]. To summarize these findings, one can notice that the choice of manufacturing technology influences the material microstructure, with direct implications for Young’s modulus and compressive strength.

**Figure 10:**
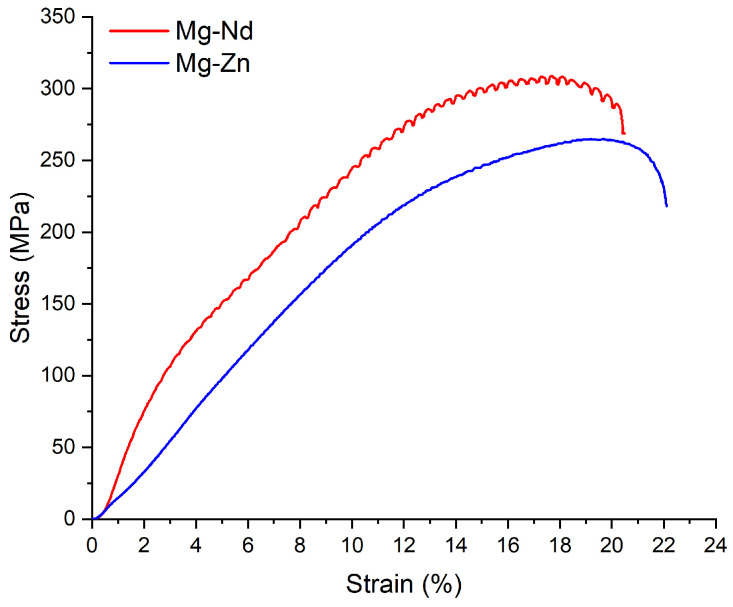
Stress and strain variations obtained after the compression test of the experimental magnesium-based alloys.

The compression tests were made on five samples prepared from each Mg-based alloy following the indications of ASTM E9-09 standard [63]. The Young’s modulus was calculated by dividing the applied stress by the corresponding measured strain in accordance with ASTM E111 standard [121]. Figure 10 presents the variations in the stress–strain curves and the compressive strength values for Mg-Nd and Mg-Zn alloys. From Figure 10, one can conclude that the strongest strain hardening occurred in the case of the Mg-Nd, while the Mg-Zn exhibited the highest deformation capacity. We could mention that the Nd and Y additions for Mg-Nd alloys generated high compressive strength. Additionally, the differences in mechanical behavior could be linked to grain size, alloying elements, precipitate formation, as well as all material microstructure characteristics. As stated before, the presence of Nd and Zn is linked to various strengthening mechanisms, and their combinations with chemical elements such as Y or Ca generate complex interactions and could modify the material properties. Both alloying elements, Nd and Zn, respectively, produce a grain refining effect.

The compressive strength is defined as the property of a material to oppose a load that tends to reduce its size. For the Mg-Nd alloy, we have obtained a higher value of about 320.21 ± 13.40 MPa compared to the Mg-Zn alloy, which was characterized by a value of 289.12 ± 12.15 MPa. It is well-known that the ultimate compressive strength of natural bone was determined to be equal to 205 MPa along the longitudinal axis and 131 MPa in the transverse direction [122,123]. The values obtained for the two investigated alloys could be considered close to those of human bone, proving that the selected materials are suitable for bone substitutes. Regarding the Young’s modulus, in the case of Mg-Nd alloy, it was determined to be a value of 59.44 ± 0.5 GPa, and for the Mg-Zn alloy, a much lower value of about 28.08 ± 0.5 GPa was obtained. The elasticity moduli obtained in our study are similar to those of human bone (trabecular bone 14.800 ± 1.40 GPa, cortical bone 20.700 ± 1.90 GPa) [124], so, as a direct consequence, the stress shielding effect, which occurs in the case of other metallic materials, is not estimated to be present for our Mg-based alloys. A one-way ANOVA analysis considering equal variances based on a confidence level of 95%, in combination with the Tukey and Fisher tests, was performed. These tests suggested that the obtained mean values of the mechanical properties are statistically significant.

Similar compressive strengths were obtained by Prakash et al. [40] for commercially available Mg-Al-Zn-Mn (AZ41) samples. The authors found a value of 337 ± 3 (MPa) for the maximum compressive strength at a strain rate of 2900 s^−1^. A much more reduced value was found in [125] for cast Mg-0.2Fe-0.8Ca (165.6 MPa). An increase of 209.7 MPa was noticed after a heat treatment at 500 °C for 4 h was applied. This observation was explained by considering the Ca dissolution in the Mg matrix due to high temperature and the grain size reduction. Teslia et al. [126] investigated the compressive strength of Mg-Zn alloys and measured a value of around 250 MPa. In comparison with sintered Mg, which had a compressive strength between 92 and 95 MPa, the increased value obtained after Zn addition was attributed to grain boundary purification processes and crystalized intermetallic presence. Su et al. [127] obtained, for Mg-0.5Ca-x (Sr, Zr, Sn), Young’s modulus values of about 45 GPa and different compressive strengths ranging between 150 MPa and 350 MPa as a function of the alloying element. Sumitomo et al. [128] determined for Young’s modulus of Mg-Al-Zn alloys a value of 40 GPa and concluded that different factors such as grain size, precipitates, and alloying elements are of utmost importance in elasticity modulus variation.

### 3.3. In Vitro Corrosion Behavior of the Experimental Samples

Upon immersion of a Mg-based alloy into aqueous solution, a corrosion process of the metallic substrate occurred, and emission of ions and atoms such as *Mg*^2+^, *OH*^−^, and *H*_2_ became possible according to Equations (1)–(3) as stated in the Introduction section [129,130]. Further, *Mg*^2+^ ions react with *OH*^−^ and generate a new compound on the substrate surface (Equation (4)). *Mg(OH)*_2_ is considered unstable in aqueous media, such as SBF, and according to Equation (5), an exchange chemical reaction occurs.

At the anode, the following *Mg* alloy dissolution reaction can be observed:

(1) $$Mg{\to Mg}^{2+}+2e^{-}$$

while at the cathode, the following occurs:

(2) $$2H^{+}+2e^{-}\to H_{2}$$

(3) $$2H_{2}O+2e^{-}\to H_{2}+2{HO}^{-}$$

Then, the metallic cations react with the hydroxyl anions, which are present in the solution, and generate magnesium hydroxide:

(4) $${Mg}^{2+}+2{OH}^{-}\to Mg{\left(OH\right)}_{2}$$

The SBF solution contains chloride ions. In the literature, it is stated that in the cases in which the *Cl*^−^ ion concentration is higher than 30 mmol/L, the *Mg(OH)*_2_ layer will react, and highly soluble *MgCl*_2_ and *OH*^−^ result [6,39,131]. For the SBF solution, the chlorine ion concentration is about 131 mmol/L [132]. It can be foreseen that bone substitute samples will be subjected to a severe corrosion process and to a rapid mass loss (Equation (5)).

(5) $$Mg(OH{)}_{2}+2{Cl}^{-}\to Mg{Cl}_{2}+2{HO}^{-}$$

**Figure 11:**
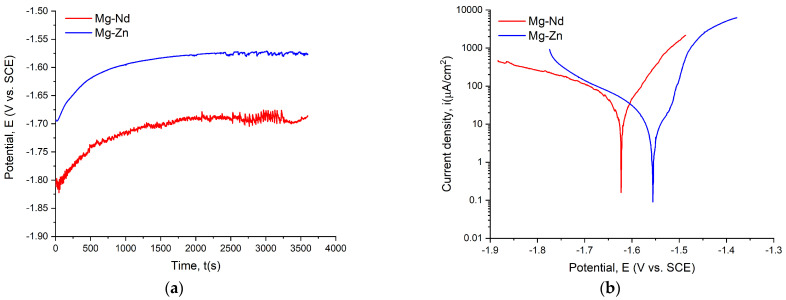
Electrochemical dependencies for Mg-Nd and Mg-Zn alloys: (**a**) OCP curves; (**b**) Tafel curves.

The open circuit potential (OCP) and Tafel curves for the investigated alloys are shown in Figure 11, while Table 4 presents the main parameters.

By using SBF as an electrolyte, it is observed that all the samples had negative values for E_oc_ and E_corr_, below −1 V. When a material exhibits an improved corrosion behavior, it presents more electropositive values of E_oc_ and E_corr_, concomitantly with electronegative values of i_corr_ and higher values of R_p_. The ASTM G59-97 standard [133] recommends that researchers compute the CR only for uncoated samples. For excellent corrosion behavior, this parameter must have reduced values.

By considering the E_oc_ value criteria, it can be observed that the Mg-Zn samples had a higher value of −1.567 V than the −1.675 V obtained for the Mg-Nd samples. These findings can be easily verified with the E_corr_ criteria, with higher values of this parameter in the case of Mg-Zn samples: E_corr_ = −1.566 V; and lower values for the Mg-Nd samples: E_corr_ = −1.620. Regarding the corrosion current density i_corr_, it can be observed that the lowest value, being equivalent to the most electronegative character, was obtained for the Mg-Zn sample (6.859 μA/cm^2^). The Tafel curves proved that the corrosion current density shifted in a more noble direction for the Mg-Zn samples, offering increased resistance to the corrosion process and supporting the data presented in Table 4. In [134], the anodic polarization curves are associated with the Mg dissolution process, while the cathodic curves are usually linked to hydrogen emission. Higher values of polarization resistance showed good corrosion resistance. In our case, the highest value was obtained for Mg-Zn (7.102 kΩcm^2^). The CR parameter clearly indicates that the Mg-Zn alloy has a significantly reduced value compared to Mg-Nd, which demonstrates better corrosion resistance, as also supported by the other criteria explained in this section.

Many studies in the literature describe the electrochemical corrosion behavior of Mg-based alloys. Zemkova et al. [82] investigated the Y and Nd additions on Mg alloys directly related to their corrosion behavior. It was observed that the Mg-Y secondary phase particles had a detrimental influence on the corrosion resistance [135,136], while in the cases in which only Y was added, it dissolved into the Mg matrix, generating an increase in the corrosion resistance [137]. In addition, the authors noticed that for Mg-4Y-3Nd alloy, the secondary Mg-(Nd, Y) phase did not produce an important galvanic corrosion effect due to a reduced potential difference of 25 mV between α-Mg and the secondary phases. Regarding the Mg-Zn-Mn-Ca alloys, some investigations showed that the secondary phase Mg_2_Ca could decrease the corrosion rate in alloys with a Zn/Ca ratio below 1.25. Unfortunately, this ratio is about 2.8 in our case, and the Ca content is lower than 0.5%. It can be concluded that in the absence of the above-mentioned phase, the α-Mg corrodes much faster due to the galvanic coupling phenomena that occurred in the material, while Ca_2_Mg_6_Zn_3_ remains in the alloy and generates an increase in the corrosion resistance in the case of an increased Zn content [134].

Electrochemical characterization is an important investigation because it permits researchers to completely understand the kinetic phenomena that usually accompany the Mg degradation process. In this way, parameters such as corrosion rate and polarization resistance can be computed, as they are crucial for analysis dedicated to highly bioactive metals.

### 3.4. In Vivo Animal Study

Mild postoperative complications, such as subcutaneous emphysema, were observed, likely associated with hydrogen gas release from magnesium alloy degradation.

#### 3.4.1. Histopathological Examination

**Figure 12:**
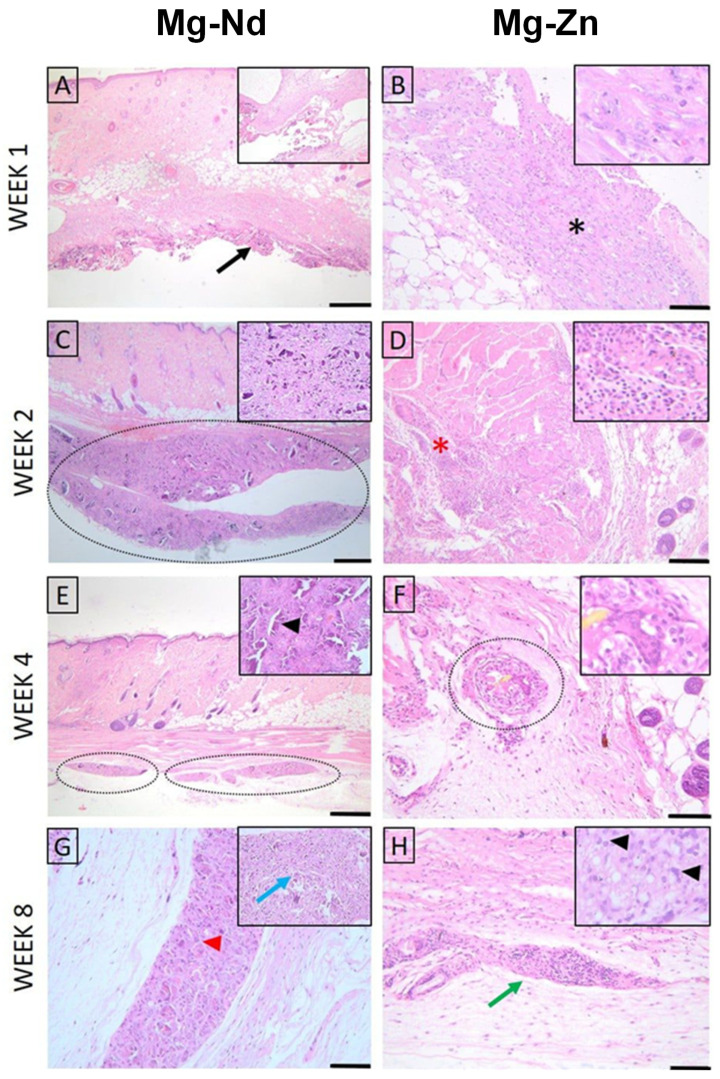
Histopathological evaluation of skin and *subcutis* lesions (Mg-Nd alloy: (**A**,**C**,**E**,**G**), and Mg-Zn alloy: (**B**,**D**,**F**,**H**)): (**A**) Minimal necrosis and mild infiltration by polymorphonuclear cells and lymphocytes, with minimal plasma cells, moderate macrophages, and mild multinucleated giant cells. The lesion is indicated by a black arrow. Inset: higher magnification of the inflammatory infiltrate; (**B**) Moderate necrosis (black asterisk) accompanied by moderate polymorphonuclear cells, minimal lymphocytes, moderate macrophages, and minimal giant cells. Inset: detailed view of necrotic tissue with inflammatory cells; (**C**) Lesion showing minimal necrosis (dotted ellipse) and mild polymorphonuclear cells, mild lymphocytes, moderate macrophages, and minimal giant cell infiltration. Inset: higher magnification highlighting inflammatory cell composition; (**D**) Area with moderate lymphocyte infiltration (red asterisk), minimal plasma cells, mild macrophages, and moderate giant cells. Inset: details of lymphocyte-predominant infiltrate; (**E**) Mild lymphocytes, macrophages, and multinucleated giant cells are observed. Multifocal inflammatory foci are marked (dotted ellipses). Inset: higher magnification of a macrophage-rich focus (black arrowhead); (**F**) Minimal lymphocytes and macrophages with moderate giant cells are seen within a perivascular lesion (dotted circle). Inset: detail showing multinucleated giant cells; (**G**) Mild lymphocytes and moderate macrophages with mild multinucleated giant cells. Minimal neovascularization and fibrosis are present. Lesion core indicated by a red arrowhead; inset shows central necrosis with inflammatory cells (blue arrow); (**H**) Moderate lymphocyte infiltration (green arrow) with minimal macrophages and giant cells. Inset: higher magnification of lymphocytic infiltrate. H&E staining; Ob. 4× (**A**,**C**,**E**), Ob. 10× (**D**,**F**,**H**), Ob. 20× (**B**,**G**); Barr 100 μm (**B**,**G**), 200 μm (**D**,**F**,**H**), 500 μm (**A**,**C**,**E**).

For both materials, at week 1, the histopathological evaluation of skin and subcutaneous lesions displayed minimal necrosis and mild infiltration by polymorphonuclear cells and lymphocytes, with minimal plasma cells, moderate macrophages, and scattered multinucleated giant cells. In the following weeks, polymorphonuclear infiltration and necrosis diminished significantly; however, mild lymphocytes, macrophages, and multinucleated giant cells were still observed in weeks 2, 4, and 8, along with minimal neo-vascularization and fibrosis (Figure 12).

The Lesion Score was higher in week 1, followed by a decreasing trend in both biomaterials; notably, Mg-Zn displayed better compatibility in all time periods (Table 5). At the end of the evaluation, both materials could be considered to have a slight to moderate irritant effect, making them suitable for further testing on the bone defect model.

**Figure 13:**
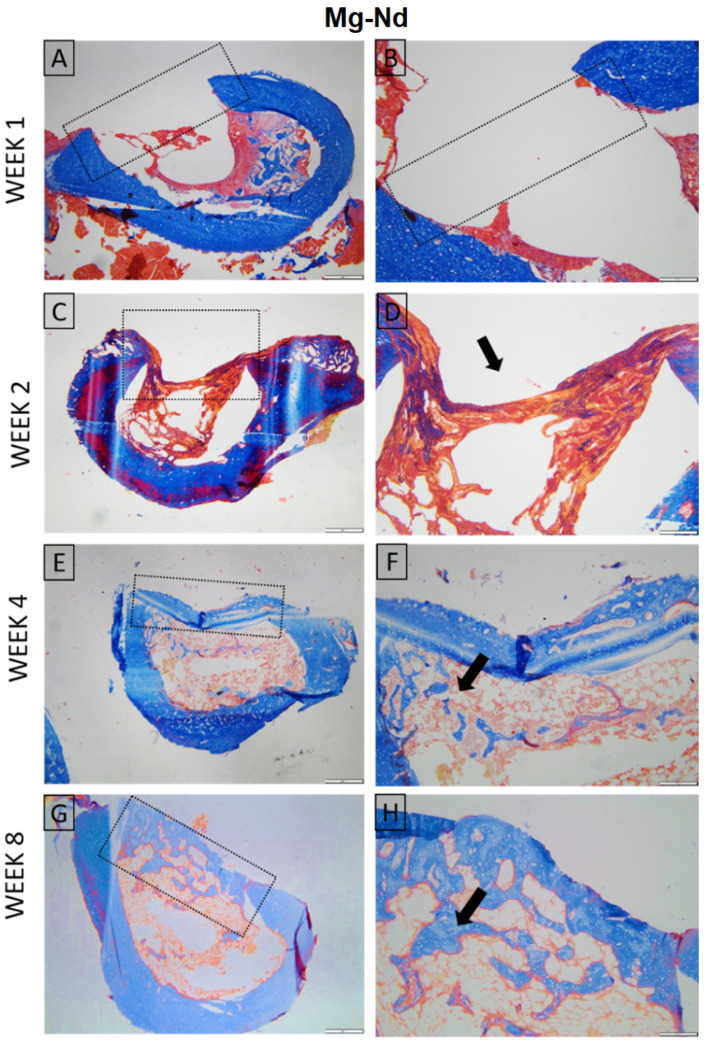
Histological aspect of the Mg-Nd group. Long bone. Week 1 (**A**,**B**) The defect is present, and no signs of inflammation or tissue regeneration are observed, consistent with the early post-injury; Week 2 (**C**,**D**) Proliferation of endosteal and periosteal tissue admixed with collagen that minimally covers the defect, without inflammation (arrow); Week 4 (**E**,**F**) Bone defect incompletely covered by compact bone; severe endosteal reaction partially separating the defect area from the bone marrow (arrow); Week 8 (**G**,**H**) Proliferation of endosteal and periosteal tissue largely covering the defect, without inflammation (arrow). Masson’s trichrome stain. Ob. ×4 (**A**,**C**,**E**,**G**) Ob. 10× (**B**,**D**,**F**,**H**). Scale bar 500 µm (**A**,**C**,**E**,**G**) and 200 µm (**B**,**D**,**F**,**H**).

**Figure 14:**
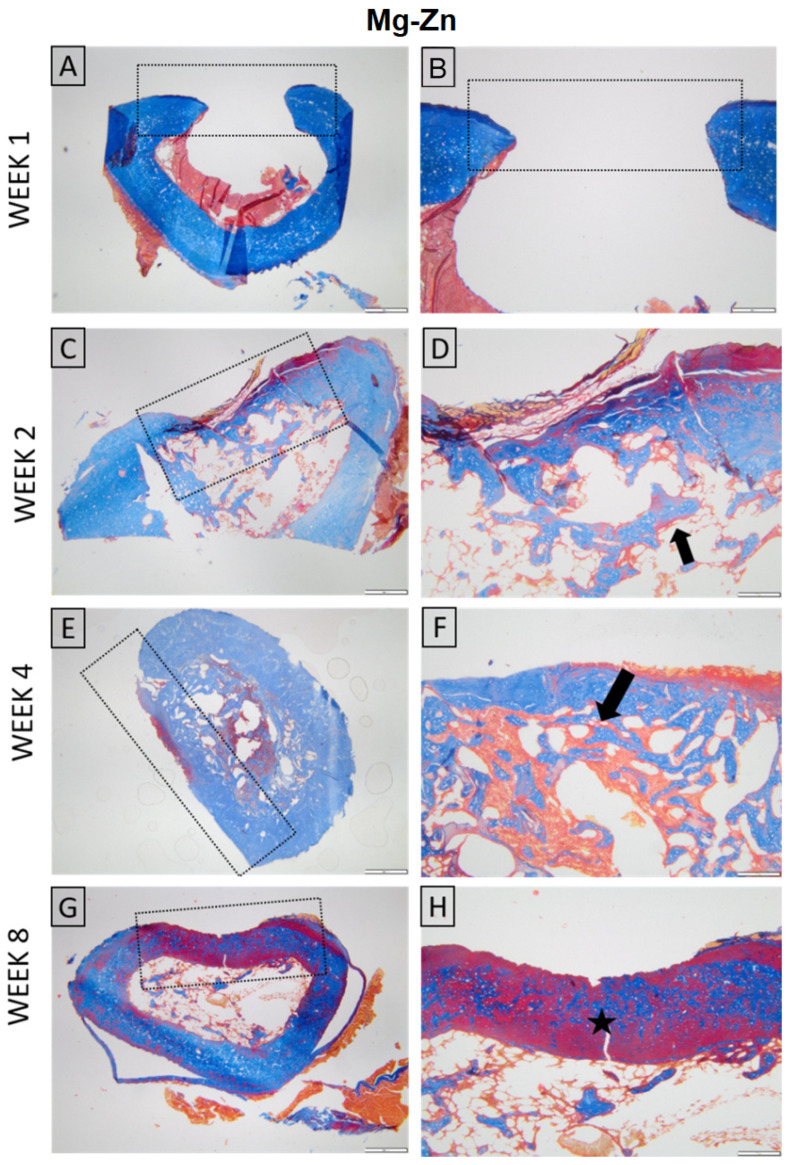
Histological aspect of the Mg-Zn group. Long bone. Week 1 (**A**,**B**) The defect is present, and no signs of inflammation or tissue regeneration are observed, consistent with the early post-injury; Week 2 (**C**,**D**) Proliferation of endosteal and periosteal tissue minimally covers the defect, without inflammation (arrow); Week 4 (**E**,**F**) Bone defect incompletely covered by bone trabecula; (arrow); Week 8 (**G**,**H**) Defect completely covered by bone tissue with a compact lamellar appearance (star). Masson’s trichrome stain. Ob. ×4 (**A**,**C**,**E**,**G**) Ob. 10× (**B**,**D**,**F**,**H**). Scale bar 500 µm (**A**,**C**,**E**,**G**) and 200 µm (**B**,**D**,**F**,**H**).

Histological evaluation showed similar early findings in both Mg–Nd and Mg–Zn groups, with the defects visible at week 1 and minimal periosteal and endosteal proliferation at week 2, in the absence of inflammation. By week 4, the Mg–Nd group displayed a pronounced endosteal reaction partially separating the defect from the marrow cavity, while the Mg–Zn group showed incomplete coverage with bone trabeculae. At week 8, the Mg–Zn group achieved complete closure with compact lamellar bone, whereas the Mg–Nd group exhibited extensive endosteal and periosteal proliferation largely covering the defect but without full lamellar organization. These results suggest that Mg–Zn alloy supports earlier maturation of bone tissue, while Mg–Nd alloy triggers a stronger endosteal response that may delay complete structural organization (Figure 13 and Figure 14).

#### 3.4.2. Micro-Computed Tomography Acquisition and Analysis

The comparative μCT analysis of bone morphogenesis between the Mg-Nd and Mg-Zn experimental samples demonstrates distinct patterns in the rate and quality of mineralized tissue growth. Over the time between week 1 and week 8, both groups exhibit a steady advancement in mineral deposition, structural maturation, and host integration, albeit at significantly differing speeds and with distinct morphologies.

**Figure 15:**
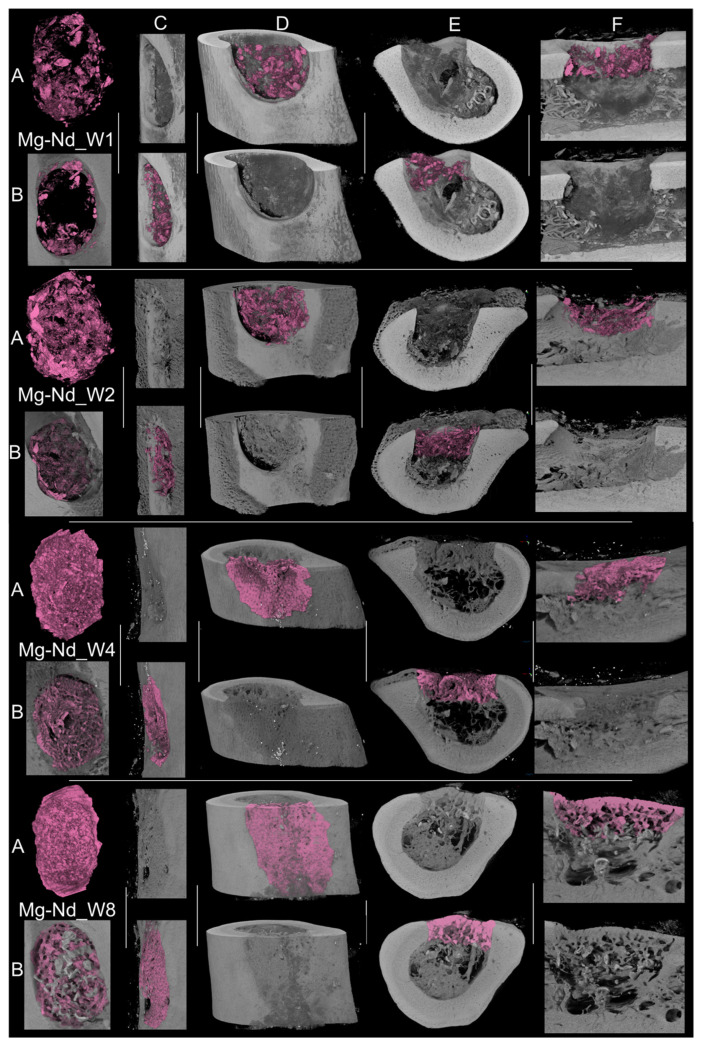
Representative 3D reconstructions and cross-sectional views of mineralized tissue within the femoral defect at multiple time points for the Mg-Nd group: (**A**) segmented bone volume; (**B**) internal view of bone tissue along the medullary canal, within the bone defect; (**C**) side-frontal view; cross-sections of (**D**) COR, (**E**) TRA, and (**F**) SAG planes illustrating bone formation and integration with the host cortex. Each view is shown with and without the segmented bone volume to highlight mineral distribution and interface continuity.

In the case of Mg-Nd experimental samples, early time points (weeks 1–2) exhibit a fragmented, low-density mineral phase within the defect area, occupying a relatively restricted volume. The *de novo* tissue exhibits porosity and an irregular morphology, characterized by scattered mineralized nuclei instead of a cohesive mass. The interfacial integration with the femoral cortex is negligible at this time, as indicated by the visible gap between the bone substitute area and the host bone edges, with some exceptions of small anchored formations interfacing the femur. The surface contact is non-continuous, and the biomaterial does not visually match the defect’s geometry. By weeks 4 and 8, there is a significant enhancement in mineral volume and density. The bone structure exhibits a more trabecular look, characterized by denser mineral clusters that increasingly resemble physiological bone architecture. The material exhibits a more uniform distribution, hence decreasing porosity. By week 8, the interface with the femoral cortex appears visually continuous, indicating integration via direct bone-to-bone bridging. The defect is filled with tissue that has not only expanded in volume but also in mineral maturity and spatial coherence (Figure 15).

**Figure 16:**
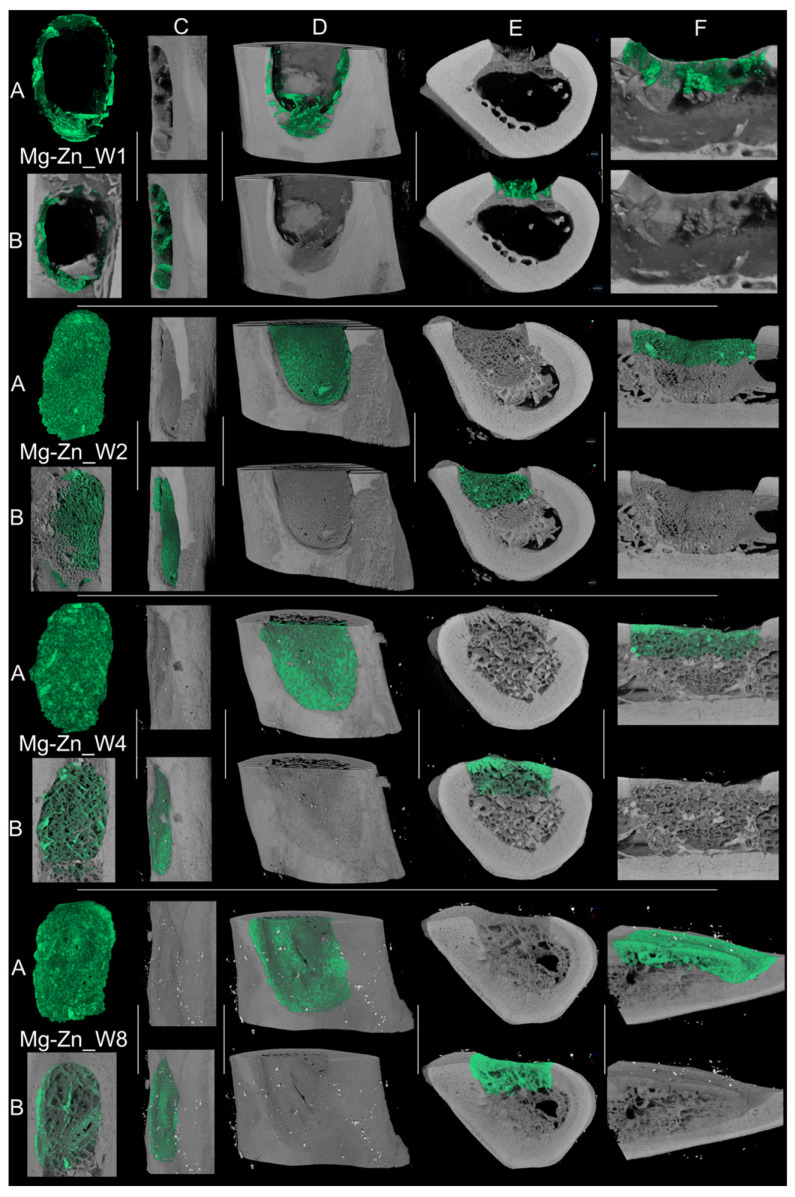
Representative 3D reconstructions and cross-sectional views of mineralized tissue within the femoral defect at multiple time points for the Mg-Zn group: (**A**) segmented bone volume; (**B**) internal view of bone tissue along the medullary canal, within the bone defect; (**C**) side-frontal view; cross-sections of (**D**) COR, (**E**) TRA, and (**F**) SAG planes illustrating bone formation and integration with the host cortex. Each view is shown with and without the segmented bone volume to highlight mineral distribution and interface continuity.

In contrast, the Mg-Zn experimental samples have elevated visible mineral density and a more uniform distribution pattern in early post-implantation images. By week 1, the mineralized phase occupies the defect volume more thoroughly, exhibiting density and a rather smooth interface with the host femur. The biomaterial adheres more closely to the defect borders, and the continuity of cortical alignment is superior. The longitudinal and cross-sectional analyses indicate a reduction in “pores” (probably an efficient natural tissue structuration) and an increase in surface regularity, implying a more efficient initial osteoconductive environment. The advancement from weeks 2 to 8 in the Mg-Zn samples is marked by both augmented mineral volume and a distinct architectural organization. The regenerated tissue exhibits increased density, with elevated greyscale intensity signifying enhanced mineral content. The junction between the newly formed bone and the surrounding femur seems progressively smoother. By week 8, the regenerated bone closely resembles the host in density and shape, indicating both mechanical and biological continuity (Figure 16). The trabecular interweaving between new and old bone is more pronounced than in the Mg-Ng group.

**Figure 17:**
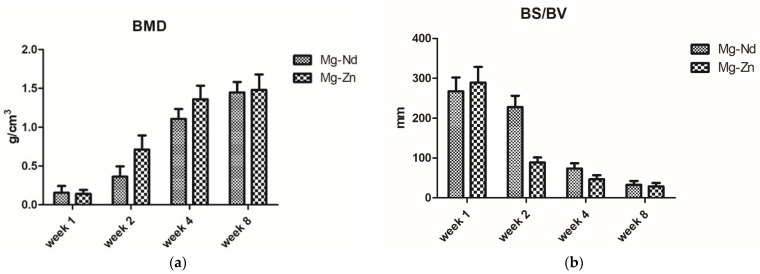
Bone mineral density (**a**) and bone specific surface (BS/BV) (**b**) at different time points.

In the comparison of both groups at all time points, Mg-Zn exhibits accelerated and more thorough mineralization, denser and more linked trabeculae, and enhanced defect conformity. The evolution seems more linear and structured, whereas Mg-Nd exhibits delayed mineral deposition, increased porosity, and less consistent interfacial bonding, especially in the initial phases (Figure 17a). The bone surface area-to-volume ratio is elevated in the early Mg-Nd samples, suggesting a more irregular structure, whereas Mg-Zn attains lower ratios more rapidly, indicative of structural consolidation (Figure 17b).

Microcomputed tomography is considered today an important tool that provides high-resolution three-dimensional images of bone, soft tissue, or other biological structures. Regarding our study, the μCT images were useful in the estimation of the mineralization capacity of the two proposed Mg-based alloys, proving their suitability as possible bone substitute materials.

Collectively, our findings indicate that Mg-Zn facilitates earlier and more cohesive bone regeneration, marked by increased mineral density, enhanced defect filling, smoother cortical transitions, and superior spatial coherence. Mg-Nd, although capable of future integration, adheres to a slower and less efficient trajectory, with mineralization and host interaction trailing behind. These disparities likely indicate changes in material bioactivity, cellular response, and scaffold architecture, eventually affecting regeneration efficacy.

#### 3.4.3. *In Vivo* Study Limitations

It is of utmost importance to emphasize that the results presented in Section 3.4, based on the Sprague Dawley rat model, may not be directly translated to human subjects due to differences in anatomical conditions, such as rat bone density, bone morphology, and metabolic rates, as well as loading conditions when analyzing bone substitutes. Regarding the anatomical differences, the human femur is significantly larger and more complex, exhibiting distinct structural properties when compared to the Sprague Dawley rat femur [138]. In addition, it is well-known that the forces and stresses acting on a bone substitute implanted in a rat’s leg cannot be applied to humans because they are significantly reduced, and only extrapolation models can be applied [139,140]. Another aspect worth mentioning is that bone remodeling processes and healing rates differ between species, and the corrosive medium inside living organisms can influence a significant portion of the alloy degradation rate, as well as the bone substitute integration mechanism [141,142]. Consequently, the primary conditions to be considered are the mechanical properties of the bone substitute, such as strength and elastic modulus, which may vary from rat to human, and it is almost impossible to predict their long-term behavior in humans [143]. Regarding biocompatibility and degradation, the breakdown process may differ, involving various responses between species [139].

It is understood that new materials are analyzed to address a clinical need and that animal models must usually be involved; however, scholars should also consider the necessity of investigating many more conditions to better understand the phenomena that occur in the human body.

As a final conclusion, these limitations narrow the application of Mg-Nd and Mg-Zn alloys in near-future clinical trials.

## 4. Conclusions

The present investigations analyzed two experimental magnesium-based alloys, from the binary systems Mg-Nd and Mg-Zn, designed for the manufacture of biodegradable structures that can be used in the treatment of bone defects. For the analyzed biodegradable structures made with biomaterials that induce osseous regeneration, the mechanical properties are not as critical as in the case of trauma implants. For structures used as bone substitutes, the most important attributes that should be analyzed are biodegradation and interaction with hard tissue, as these prove whether it is feasible for bone defect treatment.

*In vitro* corrosion tests conducted using an electrochemical approach demonstrated better corrosion resistance for the Mg-Zn alloy due to the Ca and Mn addition in a favorable amount. Also, both experimental magnesium-based alloys exhibited adequate mechanical properties with values close to those of human bone. The properties mentioned above were correlated with the microstructural characterization, directly linked to the addition of the rare earths.

Regarding the mechanical properties, the higher value of Young’s modulus obtained for the Mg-Nd alloy compared to the Mg-Zn counterparts could have a potential clinical implication by inducing local stress effects and influencing the bone remodeling process at the defect site. To address this drawback, a careful analysis and design regarding geometry, localization, and degradation rate of Mg-Nd bone substitute must be applied to achieve a mechanical equilibrium state with the natural and incremental load transfer. This fact ensures the correct bone healing mechanism, based on the simultaneous activity of osteoblasts and osteoclasts. It is advisable that patients with Mg-Nd implants should be carefully monitored to analyze the long-term effects of local stress induced in the vicinity of the bone substitute.

Good biocompatibility of both experimental magnesium-based alloys resulted from *in vivo* animal testing, a fact that supports once more the study’s initial assumption regarding the suitability of the proposed magnesium-based alloys for this kind of orthopedic need. However, the Mg–Zn alloy exhibited better hard tissue maturation, since the Mg–Nd alloy was characterized by a stronger endosteal response, which is directly linked to a delayed complete bone structural organization. The microcomputed tomography analysis revealed an accelerated mineralization process with dense and linked trabeculae being characterized by a linear evolution, in the case of Mg-Zn samples. On the other hand, for Mg-Nd samples, a delayed mineralization with a reduced interfacial bonding as well as a high porosity grade, combined with a more irregular structure, was noticed.

Based on our experimental results, we can conclude that the magnesium-based alloys, from the binary systems Mg-Nd and Mg-Zn, could be used for designing different biodegradable structures used in the surgical treatment of bone defects. Future studies must be focused on the development of biodegradable structures with patient-adapted geometries and the same long-term studies to evaluate the quality of the regenerated bone after the use of these structures.

To ensure clarity when connecting the alloy microstructure with mechanical properties and corrosion behavior, we will, in future studies, determine the statistical distribution of grain size and analyze how secondary phase distribution and their electrochemical potential modify mechanical properties, such as compressive strength and Young’s modulus, due to micro-galvanic phenomena. We want to refine our experimental methods by including a field emission electron microscope and immersion test to be confident in the observed microstructure and the obtained corrosion rates of the alloys.

In order to develop a highly efficient bone substitute, we must consider optimizations for design architecture, application of surface treatments or biocompatible coatings to reduce and control the material degradation. In this way, we will be able to address some of the limitations presented in our paper to ensure a safe clinical translation to humans.

## Figures and Tables

**Table 1:** Elemental composition of the two Mg-based alloys.

Alloy	Composition (Mass Percents %)					
	Zn	Y	Nd	Ca	Mn	Mg
Mg-Nd	0.4	0.3	2.6	-	-	Bal.
Mg-Zn	1.4	-	-	0.3	0.6	Bal.

**Table 2:** EDS chemical composition of the two Mg-based alloys.

**Element**	**Mg-Nd**					
	**O**	**Zn**	**Y**	**Nd**	**Mg**	**Total**
Weight (%)	1.56 ± 0.15	0.38 ± 0.04	0.32 ± 0.03	2.58 ± 0.12	95.16 ± 0.34	100.00
Atom (%)	2.08 ± 0.23	0.14 ± 0.02	0.09 ± 0.02	0.41 ± 0.02	97.28 ± 0.35	100.00
**Element**	**Mg-Zn**					
	**O**	**Zn**	**Ca**	**Mn**	**Mg**	**Total**
Weight (%)	0.69 ± 0.09	1.20 ± 0.13	0.35 ± 0.06	0.54 ± 0.07	97.22 ± 0.33	100.00
Atom (%)	1.02 ± 0.13	0.51 ± 0.05	0.31 ± 0.02	0.23 ± 0.03	97.93 ± 0.32	100.00

**Table 3:** Chemical composition for phase identification.

**Point**	**Mg-Nd**				
	**O**	**Zn**	**Y**	**Nd**	**Mg**
1	1.69 ± 0.16	0.21 ± 0.05	0.18 ± 0.05	1.20 ± 0.10	96.72 ± 0.31
2	2.17 ± 0.16	1.51 ± 0.13	0.00	16.45 ± 0.32	79.87 ± 0.26
3	1.70 ± 0.15	0.53 ± 0.05	0.00	2.25 ± 0.15	95.52 ± 0.28
4	1.35 ± 0.14	0.70 ± 0.06	3.02 ± 0.30	0.00	94.93 ± 0.30
5	1.87 ± 0.16	0.55 ± 0.05	0.00	2.55 ± 0.11	95.03 ± 0.29
6	1.25 ± 0.17	0.61 ± 0.10	0.00	3.94 ± 0.11	94.20 ± 0.30
7	1.28 ± 0.10	0.46 ± 0.05	0.00	2.41 ± 0.11	95.85 ± 0.30
8	1.44 ± 0.16	0.41 ± 0.05	0.00	2.26 ± 0.10	95.89 ± 0.30
**Point**	**Mg-Zn**				
	**O**	**Zn**	**Ca**	**Mn**	**Mg**
1	0.67 ± 0.09	1.2 ± 0.11	0.22 ± 0.02	0.35 ±0.07	98.23 ± 0.31
2	0.82 ± 0.10	12.71 ± 0.18	11.05 ± 0.11	0.00	75.42 ± 0.27
3	-	0.61 ± 0.05	0.12 ± 0.02	29.90 ± 0.15	69.37 ± 0.22
4	-	0.76 ± 0.09	0.19 ± 0.04	38.97 ± 0.16	60.08 ± 0.24
5	0.81± 0.09	13.73 ± 0.18	12.72 ± 0.10	0.00	72.74 ± 0.25
6	0.96 ± 0.11	15.16 ± 0.18	10.04 ± 0.09	0.00	73.84 ± 0.26

**Table 4:** The electrochemical parameters measured for experimental samples.

Sample ID	E_oc_ (V)	E_corr_ (mV)	i_corr_ (μA/cm^2^)	β_c_ (mV)	β_a_ (mV)	Rp (kΩcm^2^)	CR (mm/Year)
Mg-Nd	−1.675	−1.620	55.700	268.73	87.28	0.505	1.215
Mg-Zn	−1.567	−1.566	6.859	158.24	381.75	7.110	0.154

**Table 5:** Comparative Lesion Scores in subcutaneous implantation of the experimental alloys (ISO 10993-6).

Samples	Time	Inflammation							Neovascularization	Fibrosis	Fatty Infiltration	Subtotal	TOTAL
		Acute		Chronic				Subtotal					
		Necrosis	Polymorphonuclear Neutrophil	Lymphocytes	Plasma Cells	Macrophage	Giant Cells						
**Mg-Nd**	Week 1	1	1.5	1.5	0.5	2	1	**7.5**	1.5	2.5	0.5	**4.5**	**12**
	Week 2	0.5	1	1.5	0	1.5	0.5	**5**	2	2	0	**4**	**9**
	Week 4	1	1	2.25	0.75	2.25	0.75	**8**	1.75	1.75	0	**3.5**	**11.5**
	Week 8	0	0.25	1.5	0	2.5	1.75	**6**	1	1.25	0	**2.25**	**8.25**
**Mg-Zn**	Week 1	1.5	2.5	1	0	2	1	**8**	1	1	0	**2**	**10**
	Week 2	0	0	2	0.5	1.5	1.5	**5.5**	1	1.5	0	**2.5**	**8**
	Week 4	0	0	0.5	0	0.5	1.5	**2.5**	0	1	0	**1**	**3.5**
	Week 8	0	0	2	0.5	1.5	0.5	**4.5**	0	1	0	**1**	**5.5**

## Data Availability

The original contributions presented in the study are included in the article, further inquiries can be directed to the corresponding author.
